# Effectiveness of mucoactives (carbocisteine and hypertonic saline) in addition to usual airway clearance management with usual airway clearance management alone in acute respiratory failure (MARCH): study protocol for a multi-centre 2x2 factorial, randomised, controlled, open-label, Phase 3, pragmatic, clinical and cost-effectiveness trial with internal pilot

**DOI:** 10.3310/nihropenres.13905.1

**Published:** 2025-04-10

**Authors:** Bronwen Connolly, Naomi Dickson, Ashley Agus, Bronagh Blackwood, Mark Borthwick, Judy Bradley, Christina Campbell, Marc Chikhani, Mike Clarke, Paul Dark, Ranjit Lall, Cliona McDowell, Margaret McFarland, Michael McKelvey, Cecilia O'Kane, Brenda O'Neill, Gavin Perkins, Murali Shyamsundar, Gordon Sturmey, Clifford C Taggart, John Warburton, Barry Williams, Daniel F McAuley

**Affiliations:** 1 Wellcome-Wolfson Institute for Experimental Medicine, Queen's University Belfast, Belfast, Northern Ireland, UK; 2 Department of Physiotherapy, The University of Melbourne, Melbourne, Victoria, Australia; 3 Northern Ireland Clinical Trials Unit, Belfast, Northern Ireland, UK; 4 Oxford University Hospitals NHS Foundation Trust, Oxford, England, UK; 5 University of Nottingham, Nottingham, England, UK; 6 Centre for Public Health, Queen's University Belfast, Belfast, Northern Ireland, UK; 7 Division of Immunology, Immunity to Infection and Respiratory Medicine, The University of Manchester, Manchester, England, UK; 8 Northern Care Alliance NHS Foundation Trust Salford Care Organisation, Salford, England, UK; 9 University of Warwick Warwick Clinical Trials Unit, Coventry, England, UK; 10 Pharmacy Department, Royal Victoria Hospital, Belfast, Northern Ireland, UK; 11 Institute of Nursing and Health Research, Ulster University, Coleraine, Northern Ireland, UK; 12 Warwick Clinical Trials Unit, University of Warwick, Coventry, England, UK; 13 Regional Intensive Care Unit, Royal Victoria Hospital, Belfast, Northern Ireland, UK; 14 Independent Patient Partner, England, UK; 15 University Hospitals Bristol NHS Foundation Trust, Bristol, England, UK

**Keywords:** Acute respiratory failure, critical illness, randomised controlled trial, factorial, carbocisteine, hypertonic saline, airway clearance

## Abstract

**Background:**

Usual airway clearance management in critically ill patients with acute respiratory failure includes suctioning, humidification, use of isotonic saline, and respiratory physiotherapy techniques. Escalation to use of mucoactives occurs when secretions are difficult to clear. Use of mucoactives in clinical practice for this patient population is extensive, yet empirical and variable. Carbocisteine and hypertonic saline are the most used agents, but evidence for their effectiveness is absent or minimal. The lack of existing large-scale randomised trials comparing mucoactives to usual airway clearance management alone in critically ill patients with acute respiratory failure highlights the urgency and necessity of this study.

**Aim:**

To determine whether the use of mucoactives in critically ill patients with acute respiratory failure improves clinical outcomes and is cost effective, compared to usual airway clearance management alone.

**Methods:**

A UK multi-centre, 2x2 factorial, randomised, controlled, open-label, Phase 3, pragmatic, clinical and cost effectiveness trial with internal pilot. The target sample is 1956 critically ill adults. Participants will be equally allocated across four trial arms. All participants will receive usual airway clearance management. In three intervention groups, participants will receive either carbocisteine, hypertonic saline, or a combination of carbocisteine and hypertonic saline. In the fourth comparator group, participants will receive usual airway clearance management alone. The primary outcome is the duration of mechanical ventilation with secondary clinical, safety, and health resource utilisation outcomes. The trial will be reported in accordance with CONSORT guidelines. Ethical approval was granted by Leeds East (Yorkshire & The Humber) Research Ethics Committee (reference 21/YH/0234) on 28 ^th^ October 2021. All participants will provide written, informed consent via either Personal or Professional Legal Representatives, and subsequently directly once capacity is regained.

**Trial registration:**

Main trial: ISRCTN17683568, https://www.isrctn.com/ISRCTN17683568, 25 ^th^ November 2021

Study Within A Trial: ISRCTN16675252, https://www.isrctn.com/ISRCTN16675252, 3 ^rd^ November 2021

EudraCT Number, 2021-003763-94

## Plain Language Summary

Many patients admitted to an intensive care unit (ICU) need help to breathe from a breathing machine (ventilator). However, one problem that can occur because of being on a ventilator, is difficulty clearing secretions from the lungs. These secretions can make breathing harder if they become very thick and dry. To reduce the problem of thick secretions, the air coming from the ventilator can have moisture added to it (humidification). Other treatments can include using a suction tube to remove secretions via the breathing tube. Physiotherapists may also use techniques to help clear thick secretions. In some cases, medications called mucoactive drugs may be prescribed. We found that about one-third of patients on a ventilator in UK ICUs receive a mucoactive drug. Two common examples are 'hypertonic saline' and 'carbocisteine'. However, we do not know if these drugs really help patients with thick secretions or not.

In our trial we want to know if using one, or both, of these drugs helps with clearing thick secretions, and if as a result, this means patients spend less time on the ventilator. Patients will be allocated to one of four groups in the trial by chance. We will compare how much time patients spend on the ventilator between each group. We also want to know how safe the drugs are, and if they can improve other important outcomes during the ICU stay. Additionally, we will also look at value for money.

At the end of the trial, we will share our findings with ICUs, clinicians, researchers, and patient groups to help improve patient care. Our team includes physiotherapists, doctors, nurses, pharmacists, health economists, statisticians, former patients and family members, and others who have expertise in this type of research. Together they will ensure the trial is delivered successfully.

## Introduction

### Background

Acute respiratory failure (ARF) accounts for the majority of patient admissions to the intensive care unit (ICU) ^1– 3^ in the UK healthcare system ^4^. Invasive mechanical ventilation is the cornerstone of treatment ^2^ but increases the risk of airway secretion retention due to altered secretion rheology and impaired mucociliary clearance ^5^. Usual airway clearance management includes suctioning, humidification, use of isotonic saline, and respiratory physiotherapy techniques; it may be escalated to include use of mucoactives in patients with thick, difficult-to-clear, secretions where usual airway clearance management is insufficient ^6^. Mucoactives are a collective group of drugs, of varying mechanistic action, targeting alteration of the viscoelastic properties of mucus to promote airway clearance ^7^. National UK surveys at both ICU- and clinician-level have demonstrated that mucoactives are actively prescribed in 83% of ICUs, and at any given time, approximately 30% of patients receiving mechanical ventilation are prescribed at least one mucoactive agent; the two most common agents in use are topical (nebulised/inhaled) hypertonic saline and systemic carbocisteine ^8^. However, current practice is empirical with no supporting guidelines and wide variation in prescribing practice across ICUs and amongst clinicians, indicating considerable uncertainty about use ^6^. There is also a paucity of evidence to support effectiveness. A systematic review from 2020 investigating mucoactives in patients with ARF found the overall evidence base to be minimal, heterogenous, and with a high-risk of bias ^9^; in particular there were no data to support or refute the use of carbocisteine, and evidence was inconsistent and low-quality for hypertonic saline. We are not aware of any randomised trials that have been published since then.

The MARCH trial will deliver definitive evidence on the clinical and cost effectiveness of carbocisteine and hypertonic saline. These mucoactives have distinct mechanisms of action, which may confer differing benefits to secretion clearance ^7^. The UK critical care community also highlighted their concerns regarding the absence of evidence to guide decision making ^10, 11^, with 79% of surveyed respondents reporting the need for further research in this area and 87% of respondents being supportive of participating in a clinical trial ^8^. The lack of existing large-scale randomised trials comparing mucoactives to usual airway clearance management alone in ARF patients in the ICU, coupled with their extensive empirical use, highlights the importance of this trial to provide the evidence base needed to inform patient care.

### Rationale for the intervention

The interventions in MARCH are mucoactives, specifically carbocisteine and hypertonic saline. Both mucoactives are available commercially, relatively easy to administer, have reliable supplies and long shelf lives, and are relatively inexpensive. They are widely used in UK ICUs and represent usual clinical practice ^8^. Carbocisteine and hypertonic saline have distinct mechanisms of action. Carbocisteine, an antioxidant, is a muco-regulatory agent that regulates mucus secretion through restoring the viscoelastic properties of mucus and an anti-inflammatory effect ^7, 12^. Hypertonic saline is an expectorant mucoactive, defined as one which elicits expulsion of mucus from the respiratory tract, typically via a cough mechanism ^7^, either orally, or via the endotracheal tube in mechanically ventilated patients. Both agents are being used within their licensed range of indications, dosage, and form (according to the Summary of Product Characteristics (SPC) for carbocisteine, and the Product Instructions for Use Leaflet (PIL) for hypertonic saline) ^13^. The risk profile of using carbocisteine and hypertonic saline in this study is considered to be no higher than the risk of standard medical care, and a risk-adapted approach to their management as investigational medicinal products has been adopted ^14^. The chosen factorial study design allows for comparison of each mucoactive individually, and in combination. There is no clinical or biological rationale for, or expectation of, any interaction between the two mucoactives.

### Rationale for the comparator

The comparator group for this trial is usual airway clearance management alone, with no mucoactive delivery. All participants in the MARCH trial will receive usual airway clearance management, comprising airway suctioning, heated humidification, use of isotonic saline depending on clinician preference, and respiratory physiotherapy, which represents current practice within the NHS for critically ill patients with ARF ^6^. Carbocisteine and hypertonic saline will be delivered individually, or in combination, in addition to usual airway clearance management across the three intervention groups.

### Aim

The aim of the MARCH trial is to determine whether the use of mucoactives in critically ill patients with ARF improves clinical outcomes and is cost effective, compared to usual airway clearance management alone. Our research question is ‘In critically ill adults with acute respiratory failure, does adding mucoactives (carbocisteine, hypertonic saline, or both) to usual airway clearance management, improve clinical outcomes and is cost effective, compared to usual airway clearance management alone?’

### Objectives

***Primary objective***

To conduct a large, UK, multi-centre, pragmatic, 2x2 factorial, randomised controlled trial to determine the clinical effectiveness (for duration of mechanical ventilation) of two mucoactives (carbocisteine or hypertonic saline), or a combination of both, when compared with usual airway clearance management. In keeping with the 2x2 factorial design, the main comparisons will be the use versus non-use of carbocisteine, and the use versus non-use of hypertonic saline.

***Secondary objectives***

When compared with usual airway clearance management, to:

1.   Determine the clinical effectiveness of carbocisteine or hypertonic saline, or a combination of both, on a range of secondary clinical and safety outcomes.

2.   Estimate, in an integrated economic evaluation, the cost-effectiveness of these mucoactives.

## Protocol

This trial protocol is reported in line with the SPIRIT reporting guidelines for factorial randomised trials ^15, 16^ (available at http://doi.org/10.6084/m9.figshare.28653203)

### Patient and Public Involvement

Engagement with patients and family members has been, and remains, central to the MARCH trial. Three former patients and family members with critical illness experience initially worked with the trial team to develop the research question and trial design at the funding application stages. Their perspectives reflected their recollections of receiving and/or observing airway clearance management during critical illness and the importance of strategies, such as mucoactives, that could support that process. Discussion with our patient/family member partners contributed to determining selection of the primary outcome (duration of mechanical ventilation) as they felt strongly that coming off of the ventilator was the most important part of the critical care pathway. Other aspects of trial design informed through these discussions included consideration of eligibility criteria and which patients might receive a mucoactive intervention. Subsequently, three further former patients and family members joined the group, to form the official MARCH Patient and Family Advisory Group alongside representation from the trial team. We developed a PFAG Guidance Document to summarise key information relating to the roles and responsibilities of all PFAG members, functioning of the group (e.g. frequency of meetings, confidentiality), and details for contacting the Chief Investigator outside of meetings and the mechanism for receiving PPI remuneration. Members of our diverse PFAG have key roles in trial delivery - two members were co-applicants with the research team as part of the funding application, and a further two members sit on the Trial Steering Committee to ensure appropriate representation of, and sensitivity to, the views of patients and their families during trial oversight. Meetings with the PFAG are held quarterly and their expertise has guided further development of aspects of the trial protocol such as collection of biological samples from trial participants as part of the mechanistic study that led to parallel funding from NIHR Efficacy and Mechanism Evaluation (NIHR134567, https://fundingawards.nihr.ac.uk/award/NIHR134567), and co-production of accessible patient and family-facing materials to facilitate the consent process (including a visual summary schematic of the trial process to streamline information). The PFAG have contributed insights into optimising recruitment, in particular considering translated versions of documents and suggestions for other interactive forms of providing trial details to potential participants. As recruitment to the trial approaches completion, work with the PFAG will shift emphasis towards determining appropriate and effective dissemination routes of findings to participants and the wider lay community with, and without, experience of critical illness. We will co-develop a dissemination plan and look for opportunities to overlap this with dissemination to clinical/scientific audiences e.g. shared presentations at relevant meetings. In a co-produced and co-authored output, we plan to report our PFAG partnership work from throughout the trial using the Guidance for Reporting Involvement of Patients and the Public 2 checklist ^17^.

### Trial design

MARCH is a 2x2 full, factorial, randomised, controlled, open-label, phase 3 pragmatic, superiority, clinical- and cost-effectiveness trial, with an internal pilot, of two medicinal products (i.e. a Clinical Trial of an Investigational Medicinal Product (CTIMP)). In PICO terms:

***Population:***

Adult, critically ill patients admitted to the ICU with ARF and requiring invasive mechanical ventilation, with secretions that are difficult to clear with usual airway clearance management (as assessed by the treating clinical team)

***Intervention:***

Mucoactives (carbocisteine, or hypertonic saline, or both) in conjunction with usual airway clearance management, including suctioning, heated humidification (either active heated humidification devices, or passive heat and moisture exchangers), and respiratory physiotherapy; isotonic saline may also be used depending on clinician preference

***Comparator:***

Usual airway clearance management alone, including suctioning, heated humidification (either active heated humidification devices, or passive heat and moisture exchangers), and respiratory physiotherapy; isotonic saline may also be used depending on clinician preference

***Outcomes:***

Primary – Duration of mechanical ventilation

Secondary – Range of clinical and safety outcomes at 60 days and 6 months, cost effectiveness at 6 months

Further details of the internal pilot, embedded Study Within A Trial (SWAT), and process evaluation are reported in the Trial Protocol ( 2024_12_02_MARCH Protocol_Final V4.0, available at https://nictu.hscni.net/service/march-trial/march-trial-documents/).

### Study setting

Recruitment for the trial will take place in at least 40 adult, general ICUs, across all four UK nations, that are able to care for Level 3 critical care patients ^18^. The ICUs must provide evidence that they have a proven track record of participating in ICU research, access to the target population, a local Principal Investigator (PI) willing to lead the trial at that site with local trial team including medical, physiotherapy, and pharmacy representatives, and with clinicians in the ICU who have clinical equipoise for use of mucoactives in the target patient population and agreement to maintain trial allocation in patients randomised by their colleagues. A full list of participating sites is available on the trial website ( https://nictu.hscni.net/service/march-trial/march-study-sites/).

### Eligibility criteria

Eligibility criteria, which are the same for each factor in the factorial design, will allow enrolment of a broad and generalisable population of critically ill patients who may benefit from the therapeutic intervention, while excluding patients who may be more likely to experience an adverse reaction. Eligibility criteria are presented below with additional rationale reported in the Trial Protocol ( 2024_12_02_MARCH Protocol_Final V4.0, available at https://nictu.hscni.net/service/march-trial/march-trial-documents/).

***Inclusion criteria***

1.   Aged ≥16 years

2.   An acute and potentially reversible cause of ARF as determined by the treating physician

3.   Receiving invasive mechanical ventilation via endotracheal tube or tracheostomy

4.   Anticipated to remain on invasive mechanical ventilation for at least 48 hours

5.   Presence of secretions that are difficult to clear with usual airway clearance management (as assessed by the treating clinical team)

***Exclusion criteria***

1.   Pre-existing chronic respiratory condition receiving routine use of any mucoactive

2.   Mucoactive treatment started more than 24 hours prior to trial enrolment

3.   Known adverse reaction to either study mucoactive

4.   Treatment withdrawal expected within 24 hours

5.   Known pregnancy

6.   Previous enrolment in the MARCH trial

7.   Declined consent

8.   The treating physician believes that participation in the trial would not be in the best interests of the patient

### Interventions

***Comparator group***

Usual airway clearance management alone i.e. no mucoactive. 

***Intervention groups***

Mucoactive doses in the three intervention groups of MARCH, as follows, are those indicated in the British National Formulary ^13^. Patients in these three groups will also receive usual airway clearance management.

1. *Carbocisteine*: 750 mg three times daily, for up to 28 days, delivered systemically. Where unassisted breathing begins on Day 27 or Day 28, carbocisteine will be administered up to Day 29 or Day 30 respectively.

2. *Hypertonic saline*: 4 ml of 6% or 7% concentration, delivered via nebulisation, four times daily, for up to 28 days. Where unassisted breathing begins on Day 27 or Day 28, hypertonic saline will be administered up to Day 29 or Day 30 respectively.

3. *Carbocisteine and hypertonic saline*: as described in 1. and 2.

Both mucoactives are being used within their licensed range of indications, dosage, and form (according to the Summary of Product Characteristics (SPC) for carbocisteine, and the Product Instructions for Use Leaflet (PIL) for hypertonic saline), and represent usual clinical practice within UK ICUs ^6, 8^. For that reason, the MARCH trial has been categorised as a Type A CTIMP meaning that the risk associated with the use of both carbocisteine and hypertonic saline is considered to be no higher than the risk of standard medical care, and a risk-adapted approach to their management as investigational medicinal products has been adopted ^14^. MARCH is registered with the Medicines and Healthcare Regulatory Agency (MHRA) under their notification scheme. Further information is available in the Trial Protocol ( 2024_12_02_MARCH Protocol_Final V4.0, available at https://nictu.hscni.net/service/march-trial/march-trial-documents/). This risk-adapted approach reflects current clinical practice, ensures generalisability of trial findings, and maintains appropriate safety monitoring.

### Criteria for discontinuing or modifying allocated interventions

Study mucoactives (whether carbocisteine, or hypertonic saline, or both) will be continued until the first of the following:

1.   28 days elapse since randomisation (where unassisted breathing begins on Day 27 or Day 28, study mucoactive will be administered up to Day 29 or Day 30 respectively)

2.   First successful unassisted breathing

3.   Study mucoactive-related serious adverse event

4.   Discharge from ICU

5.   Death or discontinuation of active medical treatment

6.   Request from Legal Representative or patient to withdraw from the trial

7.   Decision from the attending ICU physician that the study mucoactive should be discontinued on safety grounds

The reason for discontinuation of treatment will be recorded on the case report form (CRF).

### Strategies to improve adherence to interventions

The administration, including any omission, of study mucoactives will be recorded in the CRF to monitor treatment compliance. Any omission of study mucoactives will not be recorded separately as a protocol deviation. Adherence to usual airway clearance management will be monitored throughout the study and, as a preventative measure, the Trial Management Group (TMG) will highlight and review any site that begins prescribing carbocisteine or hypertonic saline to participants who have been randomised to the usual airway clearance management group. Any administration of non-trial mucoactives will be recorded on the CRF. Any administration of non-trial mucoactives will not be recorded separately as a protocol deviation. Daily data collection will include study mucoactive administration and reasons for missed doses, administration of any non-trial mucoactive, and respiratory physiotherapy airway clearance management.

### Concomitant care

All aspects of intensive care management will be according to standard critical care guidelines. No part of routine ICU management is contraindicated for patients who are prescribed the study mucoactives. Patients across all four randomised groups will receive usual airway clearance management that comprises respiratory physiotherapy, airway suctioning, heated humidification, and isotonic saline (depending on individual clinician preference). Respiratory physiotherapy airway clearance management will not be protocolised but will be delivered at the discretion of treating physiotherapists based on assessment of the individual clinical need of patients ^6^. The frequency, duration, and content of treatment sessions will therefore vary among patients. However, typical airway clearance management is characterised by tailored treatment according to the specific patient presentation using a range of available techniques and evaluated using both subjective and objective outcome measures ^6^. As is the case in usual clinical practice, individual treating physiotherapists will be able to schedule their treatment sessions in combination with the delivery of the study mucoactives to optimise patient management.

### Outcomes

***Primary outcome***

The primary outcome is the duration of mechanical ventilation (in hours). This is defined (measured) as time from randomisation until first successful unassisted breathing (defined as maintaining unassisted breathing at 48 hours) or death. This outcome is one of the ‘COVenT’ core outcomes for trials of interventions intended to modify the duration of mechanical ventilation ^19^. To clarify:

i)   Unassisted breathing is defined as no inspiratory support or extracorporeal lung support

ii)   Success is defined as maintaining unassisted breathing at 48 hours

iii)   Duration includes time receiving extracorporeal lung support, invasive mechanical ventilation and non-invasive ventilation delivering volume or pressure support ventilation

iv)   Duration excludes time receiving high-flow oxygen therapy and continuous positive airway pressure

v)   Patients with a tracheostomy in situ may still achieve successful unassisted breathing

vi)   Follow-up to 60 days from randomisation

***Secondary outcomes***

Secondary clinical and safety outcomes, timing of their assessment, and measurement tools, are summarised in Table 1. The secondary outcomes of extubation, re-intubation, duration of ICU and hospital stay, all-cause mortality, and health-related quality of life represent the remaining outcomes in the COVenT core outcome set ^19^. Data contributing to the economic evaluations also represent those items recently recommended as a priority for this purpose ^20^. Clinical and safety outcomes will be measured at baseline and daily up to and including Day 28 (or the primary outcome is reached), or ICU discharge, or death, whichever comes first. Where unassisted breathing begins on Day 27 or Day 28, clinical and safety outcomes will be recorded up to Day 29 or Day 30, respectively. Participants will be followed-up to 60 days post-randomisation for the outcomes of duration of mechanical ventilation, extubation and reintubation. Health-related quality of life and all-cause mortality will be measured at 60 days, and at 6 months.

**Table 1.** Detail of secondary outcomes.

Outcome	Measurement tool, definition, method
In hospital	
Extubation	Time from randomisation to first successful extubation (success defined as remaining free from endotracheal or tracheostomy tubes at 48 hours); Censored at 60 days
Re-intubation	Event of reintubation of endotracheal tube after a planned extubation; Excludes temporary reinsertion of endotracheal tube for procedures only; Censored at 60 days
Respiratory physiotherapy input	Occurrence and frequency of airway clearance sessions; Censored at Day 28 (or the primary outcome is reached), or ICU discharge, or death, whichever comes first *
Antibiotic usage	Dose of individual agents Censored at Day 28 (or the primary outcome is reached), or ICU discharge, or death, whichever comes first *
Duration of ICU and hospital stay	Time from randomisation until patient first leaves the relevant facility or dies Censored at 6 months
All-cause mortality	Confirmation and cause of death
Safety ^#^	i) Clinically important upper gastrointestinal (GI) bleeding due to peptic ulceration confirmed on upper GI endoscopy ^i^ ii) Bronchoconstriction requiring nebulised bronchodilators ^ii^ iii) Ventilator or circuit dysfunction with respiratory deterioration ^iii^ iv) Hypoxaemia during nebulisation ^iv^ Censored at Day 28 (or the primary outcome is reached), or ICU discharge, or death, whichever comes first *
Hospital resource use	Number of days at Level of Care 0/1/2/3 Censored at 6 months
Time of consent to continue	
Health-related quality of life	EQ-5D-5L
60 days	
Health-related quality of life	EQ-5D-5L
All-cause mortality	Confirmation and cause of death
6 months	
Health-related quality of life	EQ-5D-5L
All-cause mortality	Confirmation and cause of death
Health service use since hospital discharge	Categories: care at hospital, emergency, GP surgery, health clinic, or other community setting, health care at home, medication

**Legend:** ^*^Where unassisted breathing begins on Day 27 or Day 28, data will be recorded up to Day 29 or Day 30 respectively. ICU = intensive care unit. ^#^Further definition of safety outcomes: ^i^Defined as overt bleeding on upper GI endoscopy, developing as a complication in the ICU and accompanied by 1 or more of the following features within 24 hours: a. spontaneous drop of systolic, mean arterial pressure or diastolic blood pressure of 20 mmHg or more, b. start of vasopressor or a 20% increase in vasopressor dose, c. decrease in haemoglobin of at least 2 g/dl, d. transfusion of 2 units of packed RBC or more ^30^; ^ii^Bronchoconstriction requiring nebulised bronchodilators during or up to 30 minutes following nebulisation ^31^; ^iii^Ventilator or circuit dysfunction with respiratory deterioration - This may include hypoventilation, hypoxaemia, or other signs of respiratory deterioration temporally associated with ventilator or ventilator circuit dysfunction ^32^; ^iv^Hypoxaemia during nebulisation - A drop in SpO _2_ to below 90% during or up to 30 minutes following nebulisation ^31^ requiring an increase in FiO _2_

Further detail regarding presentation of outcome data is available in the Statistical Analysis Plan (SAP) (available at https://nictu.hscni.net/service/march-trial/march-trial-documents/).

### Participant timeline

The participant timeline is presented in Table 2.

**Table 2.** Participant timeline.

	STUDY PERIOD										
	Pre-Randomisation		Intervention Period						Consent to Continue	Follow-Up	
Timepoint	Pre- Baseline	Baseline/ Day 0	Day 1	Day 2	Day 3	Days 4–6	Day 7	Days 8–28 *		Day 60 (± 14 days)	6 Months (± 14 days)
ENROLMENT											
Eligibility assessment	X										
Informed consent	X										
Enrolment/ randomisation		X									
INTERVENTIONS											
IMP administration ^#^			X	X	X	X	X	X			
ASSESSMENTS											
Baseline data		X									
Daily data			X	X	X	X	X	X			
Blood and/or sputum sampling (optional)		X			X		X				
Adverse events			X	X	X	X	X	X			
Health-Related Quality of Life (EQ-5D-5L)									X	X	X
Health Service Use											X
Mortality			X	X	X	X	X	X		X	X

**Legend:** IMP = investigational medicinal product. #: IMP = carbocisteine, or hypertonic saline, or a combination of carbocisteine and hypertonic saline. *: Where unassisted breathing begins on Day 27 or Day 28, IMP will be administered up to Day 29 or Day 30 respectively.

### Sample size

The total sample size is 1956 (489 in each of the four randomised groups). The sample size has been calculated using a median duration of mechanical ventilation of 7 days ^4, 21^ with a minimal clinically important difference of 1 day ^22^, resulting in a median duration of 6 days in the three intervention groups. This minimum clinically important value is also based on discussion with patient and family member advisors, who emphasised the importance of reducing time spent on the ventilator as a priority outcome ^23^. This median duration of mechanical ventilation and 1 day reduction treatment effect result in a hazard ratio of 0.86. Based on a log-rank test and at 90% power and a significance level of 0.05, this requires a sample size of 1856 (464 in each of the four randomised groups). Previous critical care trials have demonstrated low levels of loss to follow-up, at less than 5% ^24– 28^, and the nature of the proposed trial where all primary outcome data will be acquired whilst patients are in the ICU and identifiable to the research team, should minimise loss to follow up. Allowing loss to follow at the 5% level, requires a sample size of 1956 (489 in each of the four randomised groups).

As there is no clinical or biological rationale for, or expectation of, any interaction between the two mucoactives, the sample size has not been inflated for this purpose. This is in keeping with systematic review findings highlighting appropriate restriction of the factorial design to scenarios where treatments do not have the potential for substantive interaction ^29^.

### Recruitment

Recruitment for the MARCH trial will take place in at least 40 adult, general ICUs, across all four UK nations, that are able to care for Level 3 critical care patients ^33^. The ICUs must provide evidence that they have:

-   A proven track record of participating in ICU research

-   Access to the target population

-   Local PI willing to lead the trial at that site, with local trial team including medical, physiotherapy, and pharmacy representatives

-   Clinicians in the ICU who have clinical equipoise for use of mucoactives in this patient population and agree to maintain trial allocation in patients randomised by their colleagues

Staff must comply with the trial protocol, standard operating procedures (SOPs), the principles of Good Clinical Practice (GCP), regulatory requirements, and be prepared to participate in appropriate trial training. A training package will be provided to sites who participate in the study. A list of study sites is available from the study website ( https://nictu.hscni.net/service/march-trial/march-study-sites/).

## Trial procedures

The trial is managed by the Northern Ireland Clinical Trials Unit (NICTU).

### Consent process

All invasively mechanically ventilated patients in the ICU will be screened daily for eligibility. The outcome of the screening process and reasons for the non-enrolment of potentially eligible patients will be recorded on the MARCH trial screening log using the Screened, Eligible, Approached, and Randomised (SEAR) framework ^34^. In the absence of patient capacity to provide prospective informed consent due to clinical condition (critical illness), this will instead be sought from a Personal or Professional Legal Representative (PerLR, ProfLR respectively). The person taking informed consent will be GCP trained, suitably qualified and experienced, and have been delegated this duty on the delegation log.

A PerLR is defined as a person who is not connected with the conduct of the trial who is suitable to act as the legal representative by virtue of their relationship with the patient, and is available and willing to do so ^35^. The PerLR will be informed about the trial by the responsible clinician or a member of the research team and provided with a copy of the Participant Information Sheet (PIS). If the PerLR decides that they are willing to provide consent for their relative/friend/partner to take part, they will be asked to sign the PerLR consent form. If no PerLR is available in person or by telephone, a ProfLR may be approached to give consent. A ProfLR is defined as a doctor responsible for the medical treatment of the patient if they are independent of the study, or a person nominated by the healthcare provider ^35^. The doctor will be informed about the trial by the responsible clinician or a member of the research team and given a copy of the PIS. If the doctor decides that the patient is suitable for entry into the trial, they will be asked to sign the ProfLR consent form. 

Patients will be informed of their participation in the trial by the responsible clinician or a member of the research team, either within the ICU or acute hospitalisation period, when they regain capacity to understand the details of the trial. The responsible clinician or a member of the research team will discuss the trial with the patient and the patient will be given a copy of the PIS to keep. The patient will be asked for consent to continue to participate in the trial and to sign the consent to continue form. To inform and enable the collection of follow-up data, sites will be advised to send a letter to the participants General Practitioner (GP) to advise them of their participation in the MARCH trial. Further details are reported in the Trial Protocol ( 2024_12_02_MARCH Protocol_Final V4.0, available at https://nictu.hscni.net/service/march-trial/march-trial-documents/).

### Collection and use of biological specimens

Biological samples (sputum and blood) will be collected at sites giving agreement to do so, and with any necessary infrastructure in place to support. Consent for sample collection is a specific item detailed in the MARCH informed consent process; patients with suspected or confirmed COVID-19 disease will not have samples collected. Samples will be collected by trained staff at Baseline (Day 0), Day 3, and Day 7, and processed according to the locally available MARCH Sample Processing Guideline; where any sample cannot be collected, this will not constitute a protocol deviation. Samples will be labelled with the patient’s unique Participant Study Number, and after any local processing, will be stored at –80 °C until transfer to Queen’s University Belfast, where they will be further stored at –80 °C until analysis and beyond study completion. All necessary ethical approvals for analyses of samples, or any future study, will be secured prior to any investigation being conducted. 

### Withdrawal of consent

The ProfLR, the PerLR, or the participant may withdraw consent from the study at any time without prejudice. If consent is withdrawn this will be documented in the patient’s notes and in the CRF. The elements of the trial to be withdrawn (from the following possibilities) will be documented:

•   Mucoactive administration if ongoing

•   On-going data collection during hospital admission

•   On-going data collection following hospital discharge

•   Confirmation of vital status

If the patient or patient representative declines on-going participation, anonymised data recorded, and samples taken up to the point of withdrawal, will be included in the trial analysis unless the patient or patient representative requests otherwise. 

### Assignment of interventions: allocation

***Sequence generation***

Participants will be randomised using an automated web-based or telephone system (Centre for Healthcare Randomised Trials (CHaRT), University of Aberdeen, UK) via randomly permuted blocks in a 1:1:1:1 ratio to the four study groups. There will be stratification by recruitment centre.

***Concealment mechanism***

The randomisation sequence will be saved in a restricted section of the Trial Master File (TMF), which can only be accessed by the trial statistician and not those who enrol or assign interventions. To ensure allocation concealment, the randomisation for an individual patient will not be revealed to an appropriately trained and delegated member of the research team at their site until the patient has been recruited into the trial.

***Implementation***

After informed consent, patients will be randomised via an automated web-based or telephone system. Sites will be provided with trial specific randomisation guidelines. Randomisation will be completed by an appropriately trained and delegated member of the research team. Each patient will be allocated their own unique Participant Study Number during the randomisation process, which will be used throughout the study for participant identification on all data collection forms and questionnaires. An entry will be recorded in the patient’s medical notes noting enrolment into the study.

### Assignment of interventions: Blinding

MARCH is a prospective, randomised, open label, unblinded trial. Patients, healthcare providers, and outcome assessors, will not be blinded to the allocated intervention in this trial in order to reflect routine practice when mucoactives are (or are not) used in critical care ^36^. The trial statistician, who has no role in decision-making with regards the conduct of the trial, will be unblinded and this will also facilitate linkage with the Data Monitoring and Ethics Committee (DMEC). The remainder of the trial team will also be unblinded for the purposes of managing data collection, reviewing cases to assess protocol deviations, and to undertake pharmacovigilance duties. As the trial design is open label, there is no unblinding procedure.

### Data collection

To ensure accurate, complete, and reliable data are collected, the NICTU will provide training to site staff. All data for an individual patient will be collected and recorded in source documents and transferred onto a bespoke, web-based, electronic CRF for the study. A data dictionary, record of automatic and manual data queries, and a full audit trail will ensure data captured are consistent, reliable, and fully compliant with GCP and any other relevant regulatory requirements. For routinely collected clinical data the NHS record will be the source document. Patient identification on the CRF will be through their unique Participant Study Number, allocated at the time of randomisation. Data will be collected and recorded on the electronic CRF by the PI or designee as per the CRF submission guidelines. If the participant is transferred to another MARCH site, the PI or designated member of the site study team will liaise with the receiving hospital to ensure complete data capture as per CRF instruction. If this is not possible, the primary outcome will be collected as a minimum.

For the economic evaluation, health-related quality of life (HRQoL) will be measured using the EQ-5D-5L administered at the time of consent to continue, 60 days and 6 months. Resource utilisation data will be collected via questionnaires administered at 6 months. Where the patient has been discharged from hospital, questionnaires will be administered by post, telephone, or email by the NICTU. The participating site will provide the NICTU with contact details for the patient (including name, address and email) to enable the collection of follow-up data.

### Data management

The NICTU will provide training to site staff on trial processes and procedures including CRF completion and data collection. Source data verification (SDV) will be completed by the NICTU and will check the accuracy of entries on the electronic CRF against the source documents and adherence to the protocol. The extent of SDV to be completed is detailed in the Monitoring Plan. Quality control is implemented by the NICTU in the form of SOPs, which encompass aspects of the clinical data management process, and ensure standardisation and adherence to GCP guidelines and regulatory requirements. Data validation will be implemented, and discrepancy reports will be generated following data entry to identify discrepancies such as out of range, inconsistencies or protocol deviations based on data validation checks programmed in the clinical trial database. Following the entry of patient data into the study database, the data will be processed as per NICTU SOPs and the study specific Data Management Plan. Data queries will be generated electronically for site staff to clarify data or provide missing information. The designated site staff will be required to respond to these queries. All queries will be responded to or resolved within the study database and amended in the study database.

### Statistical methods

The primary analysis will be conducted on outcome data from all randomised patients according to the group to which they were allocated (i.e. intention-to-treat), regardless of the subsequent treatment they received. Trial results will be reported in accordance with Consolidated Standards of Reporting Trials guidance (CONSORT) ^37^. It is possible that some participants may not receive the full treatment dose, therefore a secondary per protocol analysis will be undertaken on the population who receive the complete treatment dose. Baseline characteristics, follow-up measurements and safety data, will be described using suitable measures of central tendencies; means and medians with the associated standard deviations, 95% confidence intervals, and interquartile ranges for continuous data; and frequencies and proportions for categorical data (including binary data). 

Primary outcomes for the randomised groups will be compared using a Cox proportional hazards model including site and adjusting for age and illness severity (APACHE II). For this analysis, no interaction between interventions will be assumed. Comparison for other continuous outcomes will use analysis of covariance to adjust for baseline characteristics and covariates. Comparison for binary outcomes will use generalised linear models (GLMs) as appropriate to estimate risk ratio and risk differences. 95% CI and p-value will be presented alongside the estimates. Analyses will be two-sided and tested at an *a priori* significance level of p=0.05. The factorial design permits separate testing of the effects of carbocisteine and hypertonic saline on outcomes. Although there is no biologic rationale for, or expectation that, either mucoactive will have an effect on death, a sensitivity analysis for competing risk of death will be included. Sensitivity analyses will also be included to investigate the impact of any potential interaction between the interventions on the primary analysis, to investigate the impact of contamination between the interventions, and to investigate the impact of compliance on the primary analysis. 

An independent NICTU statistician will conduct an interim analysis for the primary outcome when 60-day follow-up is available for 978 patients (half the estimated sample size), to ascertain whether assumptions made in the sample size calculations are correct. In accordance with the Haybittle-Peto stopping rule, the DMEC will be asked to make a recommendation about the future of the trial, considering the likely impact of the interim result on future practice and a p-value of less than 0.001 as "significant".

## Methods for additional analyses

Exploratory analyses for the primary outcome will be reported using interaction tests (treatment group by subgroup) and 99% confidence intervals for the following subgroups:

i)   Baseline APACHE II score

ii)   Baseline PaO _2_/FiO _2_ (PF) ratio

iii)   Pre-existing chronic respiratory condition prior to randomisation

iv)   Neurological diagnosis prior to randomisation

v)   Admission diagnostic categories; pulmonary vs. non-pulmonary

vi)   Receiving antibiotics for pulmonary infection at randomisation

The following Intercurrent Events have been identified which would prevent measurement of the primary outcome or change the interpretation of the measured primary outcome:

1.   Death prior to the timepoint at which randomised treatment is due to start

2.   (a) Hypertonic saline allocated in randomisation but not started

      (b) Carbocisteine allocated in randomisation but not started

3.   Death before successful unassisted breathing. 

4.   Transfer to another ICU before successful unassisted breathing. 

5.   Use of non-trial mucoactives 

6.   Patient withdrawal from intervention

Events 1, 2(a) and 2(b) are expected to be rare and no specific actions will be taken: analysis of these events will be by intention to treat, except for event 1 which will be handled in the same way as event 3. Event 3 will be treated as a competing risk for the primary outcome and will therefore be analysed using a hypothetical strategy. Event 5 will be dealt with using an intention to treat approach. Event 6 will also be handled using a hypothetical strategy, in which the time to unassisted breathing will be censored at the point of withdrawal and the withdrawals will be assumed to lead to missing at random data on the primary outcome. Complete follow-up should still be possible for most participants in whom event 4 occurs; if not, the hypothetical strategy used for event 6 will also be implemented.

Further details and a full description of all analyses are provided in the Statistical Analysis Plan available at https://nictu.hscni.net/service/march-trial/march-trial-documents/).

### Health economic evaluation

A full health economic evaluation will be undertaken. Although mucoactives are unlikely to impact on mortality, a reduction in the duration of mechanical ventilation may reduce ventilator-associated co-morbidities and hospital service resource use compared to usual care. The cost of a Level 3 (ICU) bed day in critical care in the UK (based on 2 to 6 organs being supported) is approximately £1680 ^38^. If the use of mucoactives results in patients coming off mechanical ventilation one day earlier and stepping down to a lower level of care, this could save more than £500 per patient with ARF (based on a Level 2 (High Dependency Unit (HDU)) bed day cost of £1136) ^38^. This is a conservative estimate of the economic saving because the patient’s overall hospital length of stay might also be reduced.

We will assess the cost-effectiveness of the treatment in the three intervention groups compared with usual care at 6 months via a cost-utility analysis. We will follow NICE methodological guidance in taking the perspective of the NHS and personal social services for the analysis ^39^. Health service use will be measured from baseline to 6 months via the CRF and a study-specific questionnaire. EQ-5D-5L response at the time of consent to continue (in lieu of a baseline measure), 60 days, and 6 months will be converted into utility scores using the UK tariff recommended by NICE at the time of the analysis. Quality adjusted life years (QALYs) will be calculated using the utilities and the area under the curve method. Recommendations have recently been published on methods for analysing economic evaluations of factorial trials and these will be used to guide analyses ^40^. In keeping with this guidance, each option in the factorial design will be treated as mutually exclusive treatments. Regression analysis with an interaction term and adjusting for baseline characteristics will estimate costs, QALYs, and net monetary benefits of each option and the incremental cost-effectiveness ratios of each option relative to the next best option will be calculated. 

Uncertainty in the data will be summarised in cost-effectiveness acceptability curves showing probability of the treatment strategies being cost-effective at different threshold levels of willingness-to-pay per QALY. Sensitivity analysis will be performed to explore impact on cost effectiveness of variations in key parameters. Further details and a full description of the analyses are provided in the Health Economics Analysis Plan (available at https://nictu.hscni.net/service/march-trial/march-trial-documents/).

### Missing data

The primary analysis will be conducted on an intention-to-treat basis. Every effort will be made to minimise missing baseline and outcome data. Standard approaches will be used to detect patterns in missing data. The level and pattern of the missing data in the baseline variables and outcomes will be established by forming appropriate tables. The likely causes of any missing data will be investigated. This information will be used to determine whether the level and type of missing data has the potential to introduce bias into the analysis results for the proposed statistical methods or substantially reduce the precision of estimates related to treatment effects. If necessary, these issues will be dealt with using multiple imputation or Bayesian methods for missing data as appropriate.

### Adverse event reporting and harms

As the MARCH trial is recruiting a population that is already in a life-threatening situation, it is expected that many of the participants will experience adverse events (AEs) and serious adverse events (SAEs). Events that are expected in this population will not be reported as an AE e.g. death, agitation, delirium, organ failure and nosocomial infections. Events that are collected as safety outcomes for the MARCH trial also will not be reported as AEs. Only SAEs that are related to the mucoactive will be reported (i.e. serious adverse reactions (SARs)). A SAE will be defined as related to the mucoactive if it is assessed as being possibly, probably, or definitely related to the mucoactive, and reported within 24 hours of the investigator becoming aware of the event. The reporting period for the trial begins upon administration of the mucoactive and ends upon termination of the mucoactive. Termination of the mucoactive will usually occur at Day 28 (or when the primary outcome is reached), ICU discharge, or death, whichever comes first. Further details are provided in the Trial Protocol ( 2024_12_02_MARCH Protocol_Final V4.0, available at https://nictu.hscni.net/service/march-trial/march-trial-documents/).

### Trial monitoring

The NICTU will be responsible for trial monitoring. The frequency and type of monitoring (on site and/or remote) will be detailed in a trial-specific monitoring plan (available on request from the NICTU) and agreed by the Sponsor. Remote monitoring activities will be arranged after enrolment of the first 1–2 patient(s) at a site, with monitoring completed within approximately 3 months and no later than 6 months of the first patient being recruited at each site. At least 1 on-site monitoring visit will be completed at those sites with a minimum of 10 patients during recruitment to the trial. Sites who fail to recruit 10 or more participants following the initial remote monitoring call will not have an on-site visit unless specifically requested by the Trial Management Group (TMG) or Sponsor. The Monitor will initially prioritise sites which have the highest recruitment rates. Additional monitoring activities (either a remote call or an on-site visit) may be triggered following central monitoring activities, at the request of the TMG or Sponsor.

Before the trial starts at a participating site, training will take place to ensure that site staff are fully aware of the trial protocol and procedures. Checks will take place to ensure all relevant essential documents and trial supplies are in place. Monitoring during the trial will check the accuracy of data entered into the CRF against source documents, adherence to the protocol, procedures and GCP, and the progress of patient recruitment and follow-up. The site PI or designee will ensure that the monitor can access all trial related documents (including source documents) that are required to facilitate the monitoring process. The extent of source data verification will be documented in the monitoring plan.

### Trial oversight

***Trial Management Group***

A TMG will be established and Chaired by the CI or Co-CI. It will comprise the CI and the Co-CI, representatives from the NICTU, and any other co-investigators who provide trial specific expertise as required at the time. The TMG will meet face to face or by online conference on a monthly basis. The roles and responsibilities of the TMG will be detailed in the local TMG Charter. Meetings will be formally minuted and a list of actions recorded and stored in the TMF. All day-to-day activity will be managed by the Trial Manager/Co-ordinators, in consultation with the CI and Co-CI.

***Trial Steering Committee***

A Trial Steering Committee (TSC) will be convened to provide oversight with respect to the conduct of the study on behalf of the Funder and Sponsor. An independent chair will lead the TSC, with at least 75% independent membership. The TSC will include the CI and Co-CI, two Patient and Public Involvement and Engagement (PPIE) representatives, and a group of experienced critical care clinicians and trialists. The TSC will meet at least annually. However, because the DMEC will meet to assess the accumulating data, the TSC may be convened to discuss issues and recommendations raised by the DMEC. Membership and roles of the TSC will be listed in the TSC Charter (available from the NICTU on request). Meetings will be formally minuted and stored in the TMF.

***Data monitoring and ethics committee***

An independent DMEC will be convened, comprising at least two independent clinicians with experience in undertaking clinical trials and caring for critically ill patients, and an independent statistician. The DMEC’s overarching responsibility is to safeguard the interests of trial participants, in particular with regard to safety, and assist and advise the TSC so as to protect the validity and credibility of the trial. The DMEC responsibilities, detailed in the DMEC Charter (available from the NICTU on request) include: monitoring the data and making recommendations to the TSC on whether there are any ethical, safety, or other reasons why the trial should not continue; considering the need for any interim analysis; advising the TSC regarding the release of data and/or information; considering data emerging from other related studies; and making recommendations to stop the trial for benefit on the basis of an effect estimate that is likely to influence decisions about the use of the relevant therapy by clinicians outside of the trial. 

The independent DMEC will meet approximately every 6 months and additional meetings can be convened in the event of any safety concerns. Separate records will be required for open and closed sessions with minutes made by the appropriate attending member of the trial team, which will be the NICTU Facilitator for the open session, and the Chair, another DMEC member, or the Trial Statistician, for the closed session. Meetings will be formally minuted and stored in the TMF.

## Ethics and dissemination

### Ethical approval

Ethical approval was granted by Leeds East (Yorkshire & The Humber) Research Ethics Committee (reference 21/YH/0234). Favourable opinion was received on 28 ^th^ October 2021. All participants will provide written, informed consent via either Personal or Professional Legal Representatives, and subsequently directly when capacity is regained.

### Protocol amendments

Management of protocol amendments and their dissemination will be undertaken by the NICTU in accordance with NICTU SOPs, and all applicable ethical and regulatory requirements. Substantial changes to the protocol will require REC and MHRA approval prior to implementation, except when modification is needed to institute an urgent safety measure to maintain patient safety. Online trial registries will be updated accordingly. Any deviations from the protocol will be documented in the CRF using the Protocol Deviation form.

### Confidentiality

In order to maintain confidentiality, all CRFs, questionnaires, study reports and communication regarding the study will identify participants by their unique Participant Study Number and initials only. Patient confidentiality will be maintained at every stage and their identity will not be made publicly available, to the extent permitted by the applicable laws and regulations.

### Data access

The agreement with each PI will include permission for trial related monitoring, audits, ethics committee review and regulatory inspections, by providing direct access to source data and trial related documentation. Each patient’s confidentiality will be maintained and their identity will not be made publicly available, to the extent permitted by the applicable laws and regulations.

### Provisions for post-trial care

The MARCH trial is recruiting in a population that is in a life-threatening situation and their vulnerability is fully appreciated. Every effort will be undertaken to protect their safety and well-being, in line with the Medicines For Human Use (Clinical Trials) Regulations 2004 and subsequent amendments, and the UK policy framework for health and social care research. The Sponsor (Belfast Health and Social Care Trust) will provide indemnity for any negligent harm caused to patients through the Clinical Negligence Fund in Northern Ireland. Queen’s University Belfast will provide indemnity for negligent and non-negligent harm caused to patients by the design of the research protocol.

## Dissemination

Trial results will be published in high quality peer-reviewed journals in accordance with the open access and threaded publication policies of the NIHR, reflecting clinical findings as well as a separate paper describing the cost-effectiveness in the NHS setting. Trial findings will also be presented at national and international meetings with abstracts available on-line. Presentation at these meetings will ensure that results and any implications are rapidly disseminated to the wider multi-professional UK intensive care community. We will actively promote the findings to maximise dissemination and uptake into future guidelines. A lay summary will be co-produced with our Patient & Family Advisory Group, who will advise on a dissemination strategy via relevant patient and family support networks. Authorship will be determined according to internationally agreed criteria for authorship ( www.icmje.org).

## Data availability

Datasets arising from the current study will be made available from the CI/co-CI via the NICTU, upon reasonable request and following discussion with the Sponsor. The study will comply with the good practice principles for sharing individual participant data from publicly funded clinical trials ^41, 42^ and data sharing will be undertaken in accordance with the required regulatory requirements. In the event of publications arising from such analyses, those responsible will need to provide the CI and Co-CI with a copy of any intended manuscript for approval prior to submission. All trial documents are available at the trial website, https://nictu.hscni.net/service/march-trial/.

## Discussion

The MARCH trial is a Phase 3, 2x2 factorial, randomised, controlled, pragmatic, clinical and cost effectiveness trial to determine whether mucoactives (carbocisteine and hypertonic saline) in critically ill patients with acute respiratory failure reduce duration of mechanical ventilation. Given the extent of prescription in clinical practice, underpinned by a paucity of high-quality evidence of effectiveness, this trial is timely and crucial for ensuring delivery of optimum patient care. Reducing uncertainty around the use of mucoactives would improve outcomes at patient and service level. If effective, they can be used more appropriately and efficiently. If ineffective, then unnecessary, or potentially harmful, delivery can be prevented with associated cost savings. Escalating pressures on ICU bed occupancy (monthly average >80% ^43^) make it a priority to determine effective treatments to reduce the morbidity associated with mechanical ventilation and consequent burden on ICU resources. A 1-day reduction in duration of mechanical ventilation across the approximately 50,000 patients admitted to ICU for ARF and receiving ventilation each year would result in significant patient, service, and economic benefits.

## Trial status

Recruitment to the trial opened on 1 ^st^ February 2022, with the first patient enrolled on 25 ^th^ February 2022. Currently (as of 31 ^st^ January 2025) 1820 patients have been enrolled, with 136 remaining to reach the sample size. Recruitment is anticipated to complete around April 2025.

## Administrative information

### Trial registration

Main trial: ISRCTN17683568, https://www.isrctn.com/ISRCTN17683568, 25 ^th^ November 2021

Study Within A Trial: ISRCTN16675252, https://www.isrctn.com/ISRCTN16675252, 3 ^rd^ November 2021

EudraCT Number, 2021-003763-94

### Protocol version

This manuscript is based on MARCH protocol: 20131DMcA-AS_v4.0 Final_02/12/2024.

## Abbreviations

AE: Adverse event; APACHE: Acute Physiology and Chronic Health Evaluation; ARF: Acute respiratory failure; BHSCT: Belfast Health and Social Care Trust; CI: Chief Investigator; Confidence interval; CONSORT: Consolidated Standards of Reporting Trials; CRF: Case report form; CTIMP: Clinical Trials of Investigational Medicinal Products; DMEC: Data Monitoring and Ethics Committee; EudraCT: European Union Drug Regulating Authorities Clinical Trials; GCP: Good Clinical Practice; GLM: Generalised linear model; GP: General Practitioner; HDU: High dependency unit; HRQol: Health-related quality of life; HTA: Health Technology Assessment; ICU: Intensive care unit; ISRCTN: International Standard Randomised Controlled Trial Number; MHRA: Medicines and Healthcare Products Regulatory Agency; NICE: National Institute for Health and Care Excellence; NICTU: Northern Ireland Clinical Trials Unit; NIHR: National Institute for Health and Care Research; PerLR: Personal Legal Representative; PF: PaO _2_/FiO _2_; PI: Principal Investigator; PICO: Population, Intervention, Comparison, and Outcome; PIL: Product Instructions for Use Leaflet; PIS: Participant Information Sheet; ProfLR: Professional Legal Representative; QALY: Quality-adjusted life year; REC: Research Ethics Committee; SAE: Serious adverse event; SAR: Serious adverse reaction; SDV: Source data verification; SEAR: Screened, Eligible, Approached, Randomised; SOP: Standard Operating Procedure; SPC: Summary of Product Characteristics; SPIRIT: Standard Protocol Items: Recommendations for Interventional Trials; TMF: Trial Master File; TMG: Trial Management Group; TSC: Trial Steering Committee

## Data Availability

No data are associated with this article. Extended data (relating to all official trial documents) are freely available on the open access MARCH trial website, https://nictu.hscni.net/service/march-trial/. Figshare: SPIRIT checklist for the MARCH trial protocol is available at http://doi.org/10.6084/m9.figshare.28653203 ^44^. Data are available under the terms of the Creative Commons Attribution 4.0 International license (CC-BY 4.0).
