# Supplementary material for: Effectiveness of mucoactives (carbocisteine and hypertonic saline) in addition to usual airway clearance management with usual airway clearance management alone in acute respiratory failure (MARCH): study protocol for a multi-centre 2x2 factorial, randomised, controlled, open-label, Phase 3, pragmatic, clinical and cost-effectiveness trial with internal pilot
Source: NIHR Open Res. 2025 Apr 10;5:30. [Version 1] doi: 10.3310/nihropenres.13905.1 (PMC12163365; doi:10.3310/nihropenres.13905.1)
Supplement: Peer review history

# Reviewer report: Reviewer response for version 1

**DOI:** 10.3310/nihropenres.15112.r35606

**Published:** 2025-06-12

**Authors:** Bairbre A McNicholas

**Affiliations:** 1 University of Galway, Galway, Ireland

This is a well written, logical protocol for an important study. The inclusion and exclusion are clearly outlined, the delivery is also sensible. Can they clarify how they will deal with cross over- i.e. for the control group where physicians feel that mucoactive is required as part of usual care? Can they clarify mucoactive related side effects as this is mentioned earlier on but its not specifically mentioned in adverse event reporting and harm.

Is the study design appropriate for the research question?

Yes

Is the rationale for, and objectives of, the study clearly described?

Yes

Are sufficient details of the methods provided to allow replication by others?

Yes

Are the datasets clearly presented in a useable and accessible format?

Yes

Reviewer Expertise:

ARDS, Intensive Care Medicine

I confirm that I have read this submission and believe that I have an appropriate level of expertise to confirm that it is of an acceptable scientific standard.

# Reviewer report: Reviewer response for version 1

**DOI:** 10.3310/nihropenres.15112.r35228

**Published:** 2025-05-14

**Authors:** Bodil S Rasmussen

**Affiliations:** 1 Aalborg University Hospital, Aalborg, Denmark

This is a well-designed pragmatic trial in invasively mechanically ventilated critically ill adult ICU patients with acute respiratory failure. The trial is preregistered. 

 The topic is clinical relevant and important as the evidence is sparse. 

 It is a phase III multicenter, 2x2, superiority, randomized, open-label, unblinded controlled trial performed in 40 ICUs in UK. The statistical analysis plan, including the power calculation is well-founded and of the highest standard.

 The primary and secondary outcomes, which also includes economic aspects are well described and relevant. 

 Patients and family members have been and will be engaged in all phases of the trial. 

 The trial will be reported following the SPIRIT reporting guidelines for factorial randomized trials. 

 Well-written and clearly presented. No specific comments.

Is the study design appropriate for the research question?

Yes

Is the rationale for, and objectives of, the study clearly described?

Yes

Are sufficient details of the methods provided to allow replication by others?

Yes

Are the datasets clearly presented in a useable and accessible format?

Yes

Reviewer Expertise:

Acute respiratory failure in critically ill patients in the ICU. Invasive mechanical ventilation. Oxygen.

I confirm that I have read this submission and believe that I have an appropriate level of expertise to confirm that it is of an acceptable scientific standard.
